# Pastoralism Can Mitigate Biodiversity Loss on Global Rangelands

**DOI:** 10.1093/biosci/biaf158

**Published:** 2025-11-18

**Authors:** David D Briske, Joris P G M Cromsigt, Jonathan Davies, María E Fernández-Giménez, Matthew W Luizza, Pablo Manzano, Rashmi Singh

**Affiliations:** Department of Ecology and Conservation Biology, Texas A&M University, College Station, Texas United States; Department of Wildlife, Fish, and Environmental Studies, Swedish University of Agricultural Sciences, Umeå, Sweden; Copernicus Institute of Sustainable Development, Utrecht University, Utrecht, The Netherlands; Centre for African Conservation Ecology, Nelson Mandela University, Gqeberha, South Africa; Independent researcher in Hereford, Herefordshire, England, United Kingdom; Department of Forest and Rangeland Stewardship, Colorado State University, Fort Collins, Colorado, United States; Natural Resource Ecology Laboratory, Colorado State University, Fort Collins, Colorado, United States; Basque Centre for Climate Change, Leioa, Spain; Ikerbasque, Basque Foundation for Science, Bilbao, Spain; Department of Liberal Arts, Indian Institute of Technology, Hyderabad, India

**Keywords:** biodiversity conservation, community-led governance, pastoralism benefits, working lands conservation, rangeland biodiversity

## Abstract

Sustainable pastoralism represents a primary strategy for supporting goals of the Kunming–Montreal Global Biodiversity Framework. Sixty-seven percent of biodiversity hotspots and 38% of key biodiversity areas globally include rangelands, but international conventions seldom recognize this vast biodiversity repository. We summarize four synergies between pastoralism and biodiversity conservation: working lands conservation, continuation of vital disturbance regimes, connectivity through transhumance corridors, and community-led governance. Actions that leverage these synergies offer critical opportunities to mitigate biodiversity loss through the creation of a vast conservation network that includes working lands and protected areas. This will require that the contemporary conservation paradigm envision pastoralists as an asset rather than a threat to biodiversity conservation and recognize grazing and fire as ecological disturbances vital to the maintenance of biodiversity. Greater inclusion of rangelands and sustainable pastoralism within global conservation frameworks has high potential to enhance attainment of global biodiversity targets.

Human activities have been diminishing Earth’s life support system at an accelerating rate since the midtwentieth century (Díaz et al. 2019, Pereira et al. 2024). Biodiversity—a critical component of this system—has also undergone substantial loss (box 1). Terrestrial ecosystems are estimated to have lost 20% of their biodiversity and global wildlife populations have decreased by 73% during the past five decades (Díaz et al. 2019, WWF 2024). A total of 705 vertebrate and 571 plant species have gone extinct since 1500 and the number of species of all taxa currently threatened with extinction is estimated to be approximately 1 million. Biodiversity loss of this magnitude has serious implications for Earth’s life support system and human well-being (Díaz et al. 2019, Pereira et al. 2024).

Rangelands are dominated by native vegetation—primarily grasses, forbs, shrubs and scattered trees—and supply diverse ecosystem services to support human well-being (Briske and Coppock 2023). They encompass multiple biomes, including deserts, grasslands, shrublands, savannas, and tundra, and represent the largest land cover type on Earth (54%, 80 million square kilometers [km^2^]; box 1; UNCCD 2024). The terms *rangelands* and *drylands* are often used broadly and interchangeably, so we have used the terminology reported in the cited references (Briske et al. 2025). However, drylands are defined exclusively on climatic criteria (aridity index < 0.65), whereas rangelands are a more inclusive category defined by vegetation cover and land use. Regardless of their designation, the vast extent and heterogeneity of these lands support diverse flora and fauna, including many species unique to these ecosystems (Lewin et al. 2024). Consequently, they provide a rich but often unrecognized reservoir of global biodiversity.

Pastoralism is the primary land use on rangelands and the most widespread land use globally (Ritchie and Roser 2019), providing livelihoods for approximately 500 million people and supplying 16% of global food production (UNCCD 2024). Pastoralism represents a lifeway based on a livestock production system that integrates economic, social, and environmental dynamics in support of human livelihoods (Nyariki and Amwata 2019), including occupational and cultural identities (Fernández-Giménez and Wilmer 2024). In the present article, we use the term *pastoralism* to reference livestock grazing or browsing on natural vegetation across extensive rangelands, often with mobile herds, in contrast to confined livestock rearing on domesticated forages and crops. Pastoral livestock have varied impacts on biodiversity, which depend on a complex set of ecological interactions (Riginos et al. 2012, Bilali et al. 2022), in combination with diverse socioeconomic and political circumstances (Fernández-Giménez and Wilmer 2024). However, pastoralism is known to have positive effects on biodiversity, even though it is often mistakenly perceived to have only negative impacts (Nori and Scoones 2023, Julián-Posada et al. 2025).

Box 1. **Glossary of key terms.**

**Biodiversity:** All living organisms on Earth encompassing the different species of plants, animals, and microorganisms, their genetic variations within each species, and the ecosystems they inhabit.

**Biodiversity hotspot:** Region of high biodiversity, including many species that are found nowhere else on Earth, that has been threatened by substantial habitat loss. Thirty-six hotspots occupy 2.5% of the Earth’s surface and support 60% of the plant, bird, mammal, reptile, and amphibian species.

**Biome:** A biological community that has formed in response to the physical environment, regional climate, and natural disturbance regimes, and that extends over a large area i.e., grasslands, deserts, tundra.

**Key biodiversity areas:** Regions that are critical for the survival of unique plants and animals and the ecological communities they include. This designation is more expansive than biodiversity hotspots with over 16,000 key areas identified, which encompass 22,000,000 km^2^.

**Governance:** Structures, processes, and mechanisms through which power and authority are exercised, decisions are made, and accountability is ensured, involving formal and informal institutions, actors, and rules.

**Land degradation:** Negative trend in land condition originating from human activity that contributes to a long-term reduction or loss of biological productivity, ecological integrity, or value to humans.

**Protected area:** Geographically defined area that is designated or regulated and managed to achieve specific conservation objectives.

**Species richness:** Total number of species in a designated area or region.

Our goal in this Forum article is to promote pastoralism as a viable strategy to mitigate terrestrial biodiversity loss on rangelands. Our specific objectives are to highlight the extent and importance of rangeland biodiversity, to describe four major synergies between pastoralism and biodiversity conservation, to identify the primary mechanisms of rangeland biodiversity loss, and to justify the need to promote pastoralism as a means of conserving global biodiversity. Greater recognition of the magnitude and societal value of rangeland biodiversity may catalyze development and implementation of more effective governance and policies supporting sustainable pastoral livelihoods to achieve these objectives. The designation of 2026 as the International Year of Rangelands and Pastoralists by the United Nations General Assembly demonstrates the importance of this goal.

## Rangeland biodiversity

The magnitude and value of rangeland biodiversity are largely unrecognized in comparison with those of tropical rainforests and coral reefs (Lewin et al. 2024). This is, in part, because of limited quantification of biodiversity throughout this expansive area, the majority of which occurs in low-income countries. Estimates of plant biodiversity have been summarized in several recent reviews (Maestre et al. 2021, Zhang et al. 2023, Lewin et al. 2024). Rangelands host a great diversity of critically endangered and highly endemic wildlife species. These include large charismatic wildlife species, especially those of east and southern African savannas, which are widely recognized globally (Holechek and Valdez 2018).

**Figure 1.**
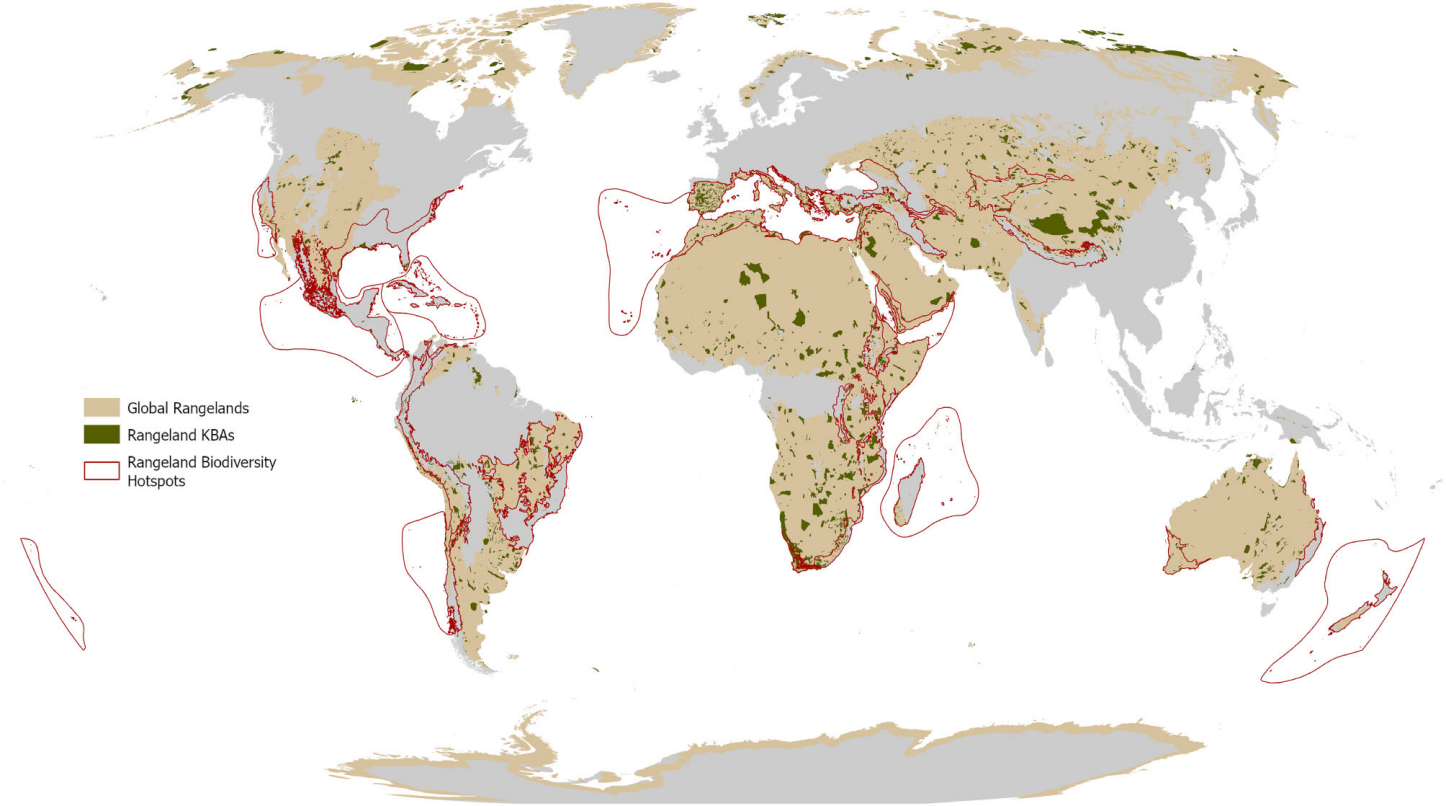
Global rangelands host numerous biodiversity hotspots and key biodiversity areas. Source: Conservation International and Critical Ecosystem Partnership Fund (2016). Map: M. Luizza, Colorado State University.

The magnitude of rangeland biodiversity is illustrated by overlaying recognized areas of high biodiversity on a map of global rangelands (figure 1; box 1). Thirty-six biodiversity hotspots are currently recognized and 24 (67%) of them include rangelands. More broadly, key biodiversity areas represent 16,232 sites occupying 22 million km^2^ globally (box 1; Key Biodiversity Areas 2024). Of this total, 6161 sites (38% of all key biodiversity areas) are found on rangelands, and they cover approximately 9.7 million km^2^ (44% of the total area of key biodiversity areas) worldwide. The Horn of Africa, southwest Australia, and Brazil’s Cerrado represent rangeland biodiversity hotspots, whereas Mongolia’s Khangain Nuruu National Park, Spain’s Sierra Nevada, and the United States’ Canyonlands Area exemplify rangeland key biodiversity areas (figure 1; Conservation International and CEPF 2016, Key Biodiversity Areas 2024). Many species found in these locations are uniquely adapted to the resource scarcity and variability of rangeland habitats and do not occur elsewhere. For example, of the estimated 14,443 plant and vertebrate species identified in the Brazilian Cerrado, over 32% are endemic to the region (Sawyer et al. 2017).

The vast biodiversity of rangelands originated from plant lineages that appeared 65 million years ago and underwent diversification in the Late Miocene and Early Pliocene 3 million to 11 million years ago (Maestre et al. 2021). This has contributed to higher functional plant diversity in rangelands compared with other regions. Plant phenotypic diversity—the range of observable traits within a population—in drylands is much greater than anticipated for environments characterized by harsh environmental conditions (Gross et al. 2024). Phenotypic diversity displayed a rapid increase (88%) at an aridity index (annual precipitation divided by evapotranspiration) of 0.7, which is similar to the value used to define drylands (0.65; box 1). Moreover, the degree of aridity required to support high phenotypic diversity decreased as grazing intensity increased, presumably in response to a decrease in vegetative cover and intensity of biotic interactions.

Biodiversity is central to the multifunctionality of rangelands, as is evidenced by the positive relationship between plant species richness and soil organic carbon, organic matter decomposition, erosion control, and forage quantity and quality (Maestre et al. 2022). A strong positive correlation has been observed between plant species richness and soil multifunctionality in less arid regions of northern China, but in more arid regions, microbial richness had a stronger correlation with soil multifunctionality (Hu et al. 2021). Continued biodiversity loss is anticipated to release substantial amounts of global carbon to create a self-reinforcing feedback loop where increasing climate change further accelerates biodiversity loss (Weiskopf et al. 2024). Consequently, mitigation strategies may be most effective if both biodiversity and climate change are simultaneously addressed (Shin et al. 2022).

## Pastoralism-Biodiversity conservation synergies

Pastoralism can maintain or in some instances enhance biodiversity through implementation of various management practices and strategies that increase landscape heterogeneity and community richness (Seid et al. 2016, Garnett et al. 2018, Julián-Posada et al. 2025). Below, we summarize four major synergies between pastoralism and biodiversity conservation. However, it is essential to acknowledge that pastoralism also has the potential to decrease biodiversity through unsustainable land-use practices, including overgrazing and human–wildlife conflict (Reid et al. 2014). These divergent outcomes of pastoralism on biodiversity underscore the urgent need to develop and implement policies and support governance institutions, at multiple levels, that will further strengthen the capacity for pastoralism to mitigate biodiversity loss.

### Working lands conservation

Protected areas currently represent the dominant approach to biodiversity conservation, but their effectiveness is limited by insufficient land area and numbers of species protected (Kremen and Merenlender 2018). Global protected areas have been estimated to provide insufficient protection for approximately 50% of 4000 species analyzed, including most species categorized as Threatened, and hundreds of mammal species (Williams et al. 2022). Similar concerns have been expressed for birds and mammals in China where protected areas are often too small and lack functional connectivity (Sun et al. 2024). It is increasingly recognized that protected areas alone are insufficient for effective biodiversity conservation without complementary conservation on working lands—lands supporting biodiversity while providing goods and services to humanity—to buffer and reduce threats to biodiversity (Kremen and Merenlender 2018).

The vast extent, heterogeneity, and ecological integrity of rangelands provides a rich reservoir of biodiversity to complement the conservation of protected areas. Rangelands represent approximately 50% of Earth’s remaining ecologically intact ecosystems (Dinerstein et al. 2019), and pastoralism has reduced biodiversity loss by limiting conversion to alternative land uses (Díaz et al. 2019). Even though rangelands have long been grazed by livestock, the majority of these lands have not been fundamentally transformed by humans and retain much of their ecological integrity (Sayre et al. 2017).

Rangeland biodiversity is highly vulnerable to loss because only 12% of rangelands are protected in comparison with 21% of other lands, which leaves many species unprotected, especially those with small ranges (Lewin et al. 2024). It has been estimated that 30% of amphibians, 7% of birds, 16% of mammals, and 27% of reptiles on rangelands do not occur in protected areas (Lewin et al. 2024). We are promoting this vast but vulnerable biodiversity repository to mitigate global biodiversity loss.

The evidence summarized above indicates that many rangeland regions arguably meet the International Union for Conservation of Nature’s definition of *other effective area-based conservation measures* (OECMs). Even though biodiversity conservation may not be a primary objective for these regions, they likely possess the potential to effectively conserve biodiversity (WCPA Task Force on OECMs 2019, Jonas et al. 2024). However, pastoralism is not explicitly mentioned within the OECM framework, and the only mention of rangelands pertains to a single candidate OECM.

### Disturbance regimes maintain biodiversity

Rangelands have evolved with multiple ecological disturbances, including grazing, fire, and drought, that occur at various frequencies and intensities and in various combinations (Bond et al. 2005, Staver et al. 2011). Pastoralism, through the continued use of grazing and fire, promotes these natural disturbance regimes to maintain biodiversity (Fuhlendorf et al. 2010, Yuan et al. 2016). In contrast, the management of protected areas often inappropriately reduces ecological function and biodiversity by suppressing these natural disturbance regimes, on the basis of the misconception that grazing and fire are unnatural, human disturbances (Abreu et al. 2017, He et al. 2019). Unethical and socially unjust displacement of Indigenous peoples for misguided conservation aims often leads to similar adverse outcomes (Goldman 2011).

Grazing has multiple direct and indirect effects on species richness and these effects strongly depend on herbivore type, grazing intensity, vegetation type, and aridity (Gao and Carmel 2020, Török et al. 2024). Our knowledge of these complex interactions is far from complete, despite multiple investigations and analyses. For example, the results of several recent meta-analyses concluded that the effects of grazing on rangeland biodiversity are often context specific and cannot be readily generalized (box 2; Yayneshet and Treydte 2015, Török et al. 2024).

Box 2. **Summary of select major analyses addressing the impacts of grazing on rangeland biodiversity.**

Livestock did not adversely affect plant or animal species richness, including carnivores, insectivores or omnivores, and the abundance of woody plants and granivores and nectarivores increased. Lack of consensus regarding the magnitude of low, medium and high grazing intensity may have partially masked grazing effects (global meta-analysis, 67 investigations; Huaranca et al. 2022).

Livestock grazing decreased plant abundance, but its impact on plant richness is complex and dependent on multiple variables. Livestock grazing decreased richness of vertebrate and invertebrate herbivores and pollinators, decreased detritivore richness, but had no effect on vertebrate predators. The potential for livestock to reduce biodiversity is greatest in mild climates with high species richness (global meta-analysis, 109 investigations; Filazzola et al. 2020)

Grazing increased plant species richness compared with grazing exclusion, especially in mesic regions. Grazing intensity also had a stronger positive effect on species richness in mesic than in arid regions, but the duration of grazing exclusion had a stronger effect on richness in arid than in mesic regions (global meta-analysis, 96 studies with 259 comparisons; Gao and Carmel 2020).

Plant species richness was less responsive to grazing than species composition, but both variables showed a modest decrease at high grazing intensities. These decreases were greater in arid than in mesic regions (meta-analysis, 48 investigations with 63 comparisons; Herrero-Jáuregui and Oesterheld 2018).

Grazing had a neutral or positive effect on plant species richness across a twenty times larger range of productivity in grasslands and savannas, with the most positive responses occurring at high productivity sites. Grazing-induced decreases in dominant plant species was considered the best predictor of grazing impacts on biodiversity (global analysis, 252 large herbivore exclosures; Koerner et al. 2018).

The consequences of communal grazing in sub-Saharan Africa are difficult to generalize because responses are strongly related to specific site characteristics, including environmental variables and soils. Nevertheless, communal grazing lands exhibited a clear reduction in species richness compared with livestock ranches and game reserves (global meta-analysis, 328 investigations; Yayneshet and Treydte 2015).

Three major conclusions emerge from these analyses of grazing impacts on biodiversity. First, they refute the widely held assumption that grazing has consistent, negative effects on biodiversity. Species richness often responds positively to increasing grazing intensity in more productive rangelands, but negatively in less productive rangelands (Bakker et al. 2006, Maestre et al. 2022). A key mechanism by which grazing increases species richness in productive areas is by reducing the competitive advantage of dominant species, thereby providing space and resources for less competitive species (Koerner et al. 2018). However, species richness in shrublands may be less affected by grazing than in grasslands because shrub dominance may be more difficult to modify depending on herbivore size and feeding behavior (Gao and Carmel 2020). Large herbivores are more likely to have positive effects on biodiversity than small herbivores, and herbivores that consume woody plants may have different effects on vegetation and biodiversity than grazers alone (Bakker et al. 2006).

Second, the spatial scale of assessment may strongly influence the response of biodiversity to grazing. The direct impacts of grazing intensity may be greater at smaller than larger scales because of the homogeneity of ecosystem variables (Hanke et al. 2014). For example, decreases in species richness in southern African rangelands were offset by greater heterogeneity of community composition and functional structure at landscape scales. Consequently, at continental and global scales the ecosystem variables of aridity and vegetation type become more important than grazing intensity and duration of grazing exclusion in determining vegetation responses.

Third, the criteria used to assess the impact of grazing on biodiversity may also influence the perceived outcomes. Species richness alone may be a less effective indicator of grazing impacts than is the diversity of functional plant types or a combination of these two variables (Hanke et al. 2014).

Fire has been a strong evolutionary force in shaping rangeland vegetation and biodiversity (Staver et al. 2011). Plant lineages in fire-prone regions often have a greater number of species than those where fire is less frequent (He et al. 2019). Therefore, fire has a lesser effect on biodiversity in arid areas than in mesic areas where fires are less frequent and intense. In more productive rangelands, the climatic potential of vegetation may not be realized because of the influence of fire and grazing (Bond et al. 2005). This enables grasslands, savannas, and shrublands to occur in climatic zones capable of supporting forests. These regions have a high fire frequency, which suppresses tree establishment and promotes the dominance of grasses and other fire- and grazing-tolerant plant species. The response of vertebrate richness to fire is less clear with both local and regional responses being context dependent (Pastro et al. 2014).

Herbivores can further influence fire regimes by altering vegetation composition and the availability of fine fuels needed to support frequent ground fires. The successive occurrence of fire and grazing is recognized to produce different ecological outcomes than fire or grazing alone (Fuhlendorf et al. 2009). The coupling of fire and grazing effectively limits shrub encroachment and creates highly diverse vegetation patterns, which contribute to high biodiversity, especially for birds and mammals (Fuhlendorf et al. 2010, Mndela et al. 2025).

The use of fire as a pastoralist management tool occurs in many countries, including in east Africa, but a comprehensive assessment of the extent of fire use is unavailable (Butz 2009). Fire is used to promote new forage growth, suppress undesirable vegetation, and minimize damaging wildfire (Seid et al. 2016). For example, in the Spanish Pyrenees, artisanal burning by pastoralists was practiced for centuries to prevent shrub encroachment in mountain grazing areas (Guadilla-Sáez et al. 2019). However, burning was prohibited following establishment of a national park, contributing to woody plant expansion and loss of landscape heterogeneity and plant species richness. Greater insight is needed into the impact of pastoralist fire management on biodiversity and the merit of local and Indigenous knowledge regarding fire management (Butz 2009).

In the absence of fire, woody plant encroachment decreases the richness of grassland specialists, and the rate of decline is dependent on the intensity of woody encroachment (Wieczorkowski and Lehmann 2022). For example, 30 years of fire exclusion in the Brazilian Cerrado increased tree density and canopy cover fourteenfold (Abreu et al. 2017). Correspondingly, species richness of savanna specialist plants and ants decreased by 67% and 86% respectively, over this period. Many rangeland ecosystems are similarly dependent on fire to maintain ecological function and biodiversity (Bond et al. 2005, Staver et al. 2011).

Fire suppression is widely implemented out of concern for loss of life and property, and in many cases, it is enforced by sociocultural norms and policies (He et al. 2019). However, these adverse outcomes are often increased by fire suppression, which contributes to the accumulation of fuels and increased fire intensity (He et al. 2019). In contrast, traditional fire management of montane heathlands by pastoralists in Ethiopia’s southern highlands has reduced the risk of larger high-intensity fires through the creation of fire breaks and has produced a mosaic of vegetation of different age classes (Johansson et al. 2012).

### Connectivity through transhumance corridors

Long-distance movement of both wild and domestic herbivores facilitates multiple ecological processes that support biodiversity. Transhumance corridors, also termed *drover’s roads* or *stock driveways*, represent designated herding routes that pastoralists have developed in response to various biophysical and sociocultural considerations (Manzano Baena and Casas 2010). Some of these routes are ancient, dating back to medieval times, and they exist on multiple continents. A well-known example is the 600-kilometer-long Conquense Drove Road, which pastoralists use annually to move sheep between mountainous and lower elevation rangelands in southern Spain. The importance of migratory corridors has led the Economic Community of West African States to regulate cross-border movements of livestock by defining corridors and promoting peaceful coexistence between migratory pastoralists and local communities (Timpong-Jones et al. 2023).

Livestock movement along these corridors serves multiple purposes, including migration between summer and winter pastures, distinct wet and dry season grazing areas, and occasionally to avoid drought and diseases (Manzano Baena and Casas 2010, Timpong-Jones et al. 2023). These corridors may extend for long distances, often crossing national borders, and include grazing areas, water sources, and infrastructure for holding livestock. Although extensive networks of corridors existed previously, their use has been declining as mechanized livestock transport has increased (Manzano Baena and Casas 2010).

Transhumance corridors promote biodiversity through two distinct mechanisms: the creation of structural heterogeneity and a pathway for rapid plant and animal movement. For example, corridors within agricultural regions or forests, which are similar in composition and structure to the surrounding rangelands, create habitat heterogeneity capable of supporting a more diverse flora and fauna (Azcárate et al. 2012). However, these benefits are scale dependent in that heterogeneity and species richness increase at local scales, but at larger scales, their structure and composition remain relatively similar.

These corridors are recognized to increase species richness in a wide array of organisms, including plants (Azcárate et al. 2012), ants (Hevia et al. 2013), and other insects (Ubach et al. 2023), including pollinators (Hevia et al. 2016). Wild bee richness and abundance was higher near drove roads in Spain, which serve as reservoirs for wild bee pollinators in adjacent agricultural landscapes (Hevia et al. 2016). Similarly, ant species richness was higher on an active drove road than on abandoned drove roads in Mediterranean agricultural regions of Spain (Hevia et al. 2013). Transhumance along elevation gradients in the eastern Pyrenees was found to maintain the richness of both plant and insect species. Summer grazing by cattle in subalpine grasslands increased butterfly richness compared with ungrazed grasslands, especially in the late summer (Ubach et al. 2023). Ungrazed rangelands became dominated by grasses, which reduced the number of nectar-producing plants capable of supporting butterflies. This indicates that the timing of grazing may be as important as the intensity of grazing in promoting biodiversity. Transhumant livestock are an important food source for rare vultures in Spain and elsewhere, leading conservationists to advocate for the continuation of this practice (Arrondo et al. 2023).

Livestock serve as effective vectors for seed dispersal, often over large distances. Seeds may be transported on the surface of animals in hair and on hooves (epizoochory) or ingested and subsequently excreted (endozoochory). For example, over 8500 seeds of 85 vascular plant species have been found in the fleece of individual sheep in grasslands of southwest Germany (Fischer et al. 1996). Experiments conducted in western Spain showed that sheep transport the seeds of multiple plant species distances of 400 kilometers in a 28-day journey (Manzano and Malo 2006). The persistent attachment of seed to the fleece during this journey, ranged from 5%–47% of the initial seed population, dependent on plant species. Transhumant livestock serve as an effective vector of long-distance seed dispersal in support of biodiversity maintenance and rangeland restoration. However, livestock may also disperse invasive and undesirable species, which may reduce native biodiversity (Kneuper et al. 2003, Kebede and Coppock 2015).

### Community-led governance fosters biodiversity

Sustainable pastoral practices and land-use strategies depend on effective institutions; the formal or informal rules, norms, policies, or laws that govern how people interact with the environment and one another; and their implementation through good governance (Bennett and Satterfield 2018). Pastoralist communities have rules regarding the timing of grazing on communally managed rangelands in systems as diverse as the Spanish Pyrenees (Fernández-Giménez and Fillat Estaque 2012), Mongolia (Fernández-Giménez 2002, Allington et al. 2024), and Indian Himalaya (Singh 2022). In Morocco’s High Atlas Mountains, the institution of pasture *agdal* maintains grazing reserves with strict opening dates after plants set seed to maintain forage productivity and support plant biodiversity (Dominguez et al. 2012). These rules enforce seasonal mobility, allow pastures to rest and recover, and maintain forage for use in the dormant season and during drought, which supports diverse plant communities and sustainable pastoral livelihoods. For example, households that made specialized fall and winter moves (*otor*) in addition to regular seasonal migrations or that set aside grazing reserves had greater species richness and functional plant diversity than pastures grazed by comparable households that did not use these practices, across Mongolia’s four major ecological zones (Fernández-Giménez et al. 2018).

Pastoralists’ rights to communal grazing land and the ability to move within and between seasonal grazing areas are essential to promote synergies between pastoralism and biodiversity conservation. Consequently, pastoralists’ collective rights to land and mobility require greater recognition and promotion by national and international institutions. Common property arrangements, where pastoralist groups hold rights to use and manage grazing lands collectively, allow for greater mobility and flexibility than individualized or private property systems, which often lead to rangeland fragmentation and block migration pathways for both livestock and wildlife (Western et al. 2009, Notenbaert et al. 2012). National laws that support the persistence of communal grazing lands include Mongolia’s Law on Land and Constitution, which prohibits the privatization of rangeland. Pastoral mobility also relies on continued access to drove roads, as is exemplified by Spain’s Drove Road Law, which protects pastoralists’ rights to move long distances using the public drove road network (Azcárate and Hevia 2023).

**Figure 2.**
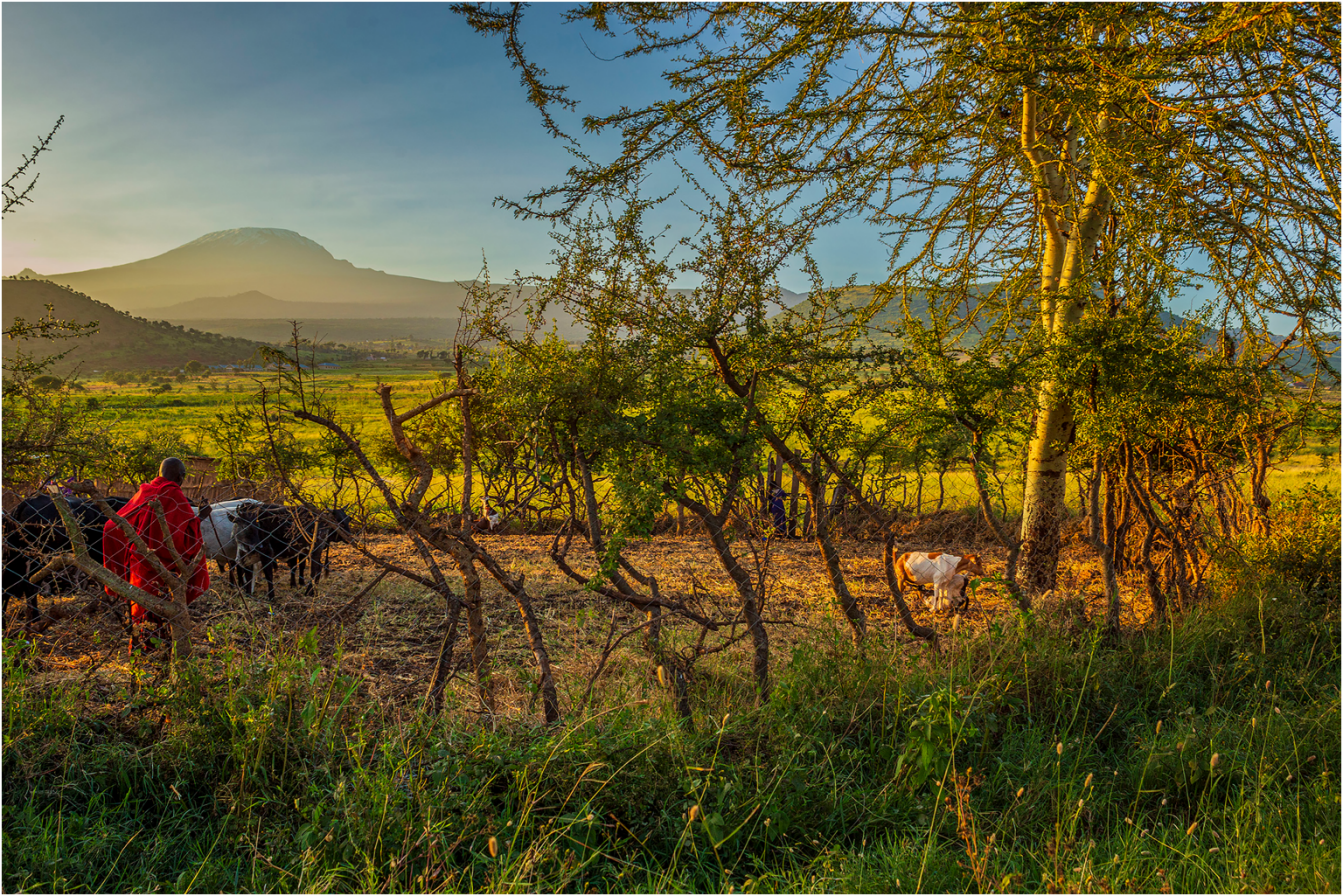
A living wall in Tanzania’s Maasai Steppe that has been codesigned by local communities and African People and Wildlife to minimize pastoralist livestock losses to predation. Approximately 2100 have been completed as of 2024. Photograph: Emmily Tunuka, African People and Wildlife.

The critical importance of local and Indigenous-group-managed areas for biodiversity conservation and human well-being is increasingly recognized by nations and biodiversity advocates (Garnett et al. 2018). Community-based and collaborative conservation efforts, such as African wildlife conservancies, which sometimes engage customary institutions to achieve conservation goals, offer an alternative to state-designated protected areas that often exclude local peoples (box 3, figure 2). Although research has rarely assessed the environmental outcomes of these alternative approaches (Galvin et al. 2018), when measured, outcomes have mostly been positive (Wilkins et al. 2021). More robust and coherent empirical research on the links between governance and conservation outcomes is needed (Ayambire et al. 2024), especially in rangelands, which have received much less attention than other land categories (Johnsen et al. 2019). Greater recognition and support for pastoralist community-led governance may provide more effective strategies to mitigate global biodiversity loss.

Pastoral livestock is often associated with adverse consequences on wildlife, but these perceptions are largely based on faulty inference and anecdote, rather than scientific evidence (Niamir-Fuller et al. 2012, Riginos et al. 2012). For example, wildlife populations in Kenya have shown large decreases (1977–2016), and those decreases have occurred concurrently with increasing livestock numbers (Ogutu et al. 2016). However, these reductions were interpreted to have multiple causes, including human population growth, land fragmentation, human–wildlife conflict, antiquated policies and institutions regulating wildlife conservation, and increasing climate change and variability, in addition to pastoral livestock.

Competition between livestock and wildlife species can be effectively managed in most cases, and the potential exists for synergistic interactions to occur among them. For example, pastoralism was found to be critical to maintenance of bird richness in east African savannas (Gregory et al. 2010). The use of fire had important short-term effects, whereas abandoned bomas (i.e., livestock enclosures) had long-term positive effects on bird richness. Habitat quality for snow leopards (*Panthera uncia*) can be enhanced by the presence of intermediate livestock densities in the Indian Himalaya (Sharma et al. 2015). Moreover, community-based initiatives have been effectively implemented to decrease livestock–predator conflict in Africa (box 3, figure. 2).

Box 3. **Community-driven initiatives mitigate livestock–predator conflict in Africa.**

Conflict between wildlife and livestock presents key challenges to biodiversity conservation in Africa. Negative interactions involving predators are especially potent and have major implications for sustainable pastoral livelihoods and safety of rural communities. Initiatives to mitigate this conflict have been developed by local conservation NGOs in concert with pastoral communities and they have proven effective. The “Lion Guardians” program in southern Kenya draws on cultural values and knowledge of Maasai pastoralists to mitigate livestock–predator conflict and monitor predator species across communally owned group ranches. The program leverages the critical role of traditional leadership with protection and rapid response to prevent and mitigate livestock depredation through community-led monitoring. The program provides a sense of lion ownership within the community and has resulted in a near-total cessation of lion killings over an 8-year period in each area where it was implemented (Hazzah et al. 2014). The resultant increase in lion mobility has facilitated “corridors of tolerance” that allow for critical connectivity across areas where lions are unprotected (Dolrenry et al. 2020). Similar community-based programs exist in Tanzania, including the “Warriors for Wildlife” program where Maasai pastoralists monitor fortified livestock enclosures (*bomas*) called *living walls*. This program has resulted in a near 100% efficacy in preventing nighttime attacks on Maasai livestock by several carnivore species (Lichtenfeld et al. 2015). Initiatives that connect cultural values and pastoral knowledge with biodiversity conservation goals, in concert with continued innovation in mitigation interventions (e.g., reusable bomas, virtual fencing, artificial eyespot application), can support resilient rangelands for both people and wildlife. In contrast, when the knowledge and needs of pastoralists are ignored, biodiversity conservation policies can exacerbate human–wildlife conflict and perpetuate hostility toward conservation efforts. See figure 2.

## Threats to biodiversity

Land use, climate change, species overexploitation, invasive species, and pollution have been identified as the primary causes of biodiversity loss globally (Mazor et al. 2018). Climate change may become a more important driver of biodiversity loss than land-use change by midcentury, but considerable uncertainty exists because of time lags associated with the impacts of both drivers (Pereira et al. 2024). Drylands are projected to expand in response to accelerating climate change, and increasing aridity will be accompanied by rapid human population growth, especially in Africa (Huang et al. 2015). Rangelands experienced urban expansion (3.8%) at a slightly higher rate than the global average (3.5%) between 1992 and 2016, which encroaches on ranges of threatened species, with mammals being most severely affected (Ren et al. 2022).

Projections for increasing aridity create the potential for further degradation, with adverse effects on biodiversity, soil stability, and plant and animal productivity (box 1; Petz et al. 2014, Maestre et al. 2022). However, increasing vegetation cover—dryland greening derived from satellite imagery—has been observed on 40% of drylands in the past three decades despite projections for increasing aridity, and it is anticipated to continue through the middle of the century (Burrell et al. 2020, Zhang et al. 2024). The occurrence of dryland greening is associated with increased atmospheric carbon dioxide concentrations, land-use change and climate-induced shifts in temperature and precipitation. Collectively, these drivers may limit dryland biodiversity loss in the near term.

Dryland degradation presents a critical current and future threat. Unfortunately, the magnitude of the threat is uncertain because of its varied interpretation, multiple causal mechanisms, and lack of consensus regarding its extent (Gibbs and Salmon 2015). A recent report from the United Nations Convention to Combat Desertification indicated that approximately 50% of global rangelands have undergone degradation, but other assessments range from 20% to 70% (UNCCD 2024). This uncertainty makes it difficult to link degradation with biodiversity loss because the magnitude of loss and its potential for recovery are often unknown. In Mongolia, for example, assessments suggest that approximately 65% of the land area has been degraded. However, only about 10% of the area is likely to have been irreversibly changed, with the remainder showing varying degrees of structural and functional change (Jamsranjav et al. 2018). It is critical that the dominant conservation focus on protected areas with minimal human activity does not obscure the potential for working lands, which may have undergone some degradation, to mitigate biodiversity loss (Kremen and Merenlender 2018).

Biodiversity loss is not an inherent outcome of pastoralism per se, as is evidenced by the sustainability of this land use for millennia. Changing sociopolitical circumstances that decrease pastoral land access by conversion to alternative land uses and the restriction of pastoral mobility have contributed to the adverse outcome of pastoralism (Krätli et al. 2013, Petz et al. 2014, Nori and Scoones 2023). For example, population growth in rural areas is directly associated with increasing cropland expansion to support food security (Li et al. 2021). The scarcity of productive arable land contributes to the conversion of increasingly marginal land that often supports high biodiversity and a rapid decline in crop productivity following initial conversion incentivizes the conversion of additional land. Agricultural conversion frequently targets key resource areas for both livestock and wildlife, which has a disproportionately larger impact than the land area converted, especially in the dry season and during drought (Illius and O’Connor 1999).

Afforestation programs designed to mitigate climate change by removing carbon from the atmosphere represent a major threat to rangeland biodiversity. Rangelands are targeted for afforestation because the ecosystem services that they provide, including an important reservoir of both biodiversity and carbon, are unrecognized and undervalued (Briske et al. 2024, Parr et al. 2024). Experimental evidence indicates that rangeland afforestation possesses minimal potential for additional carbon storage but that it has high potential to adversely affect local communities and to reduce ecosystem services that are vital to society.

**Figure 3.**
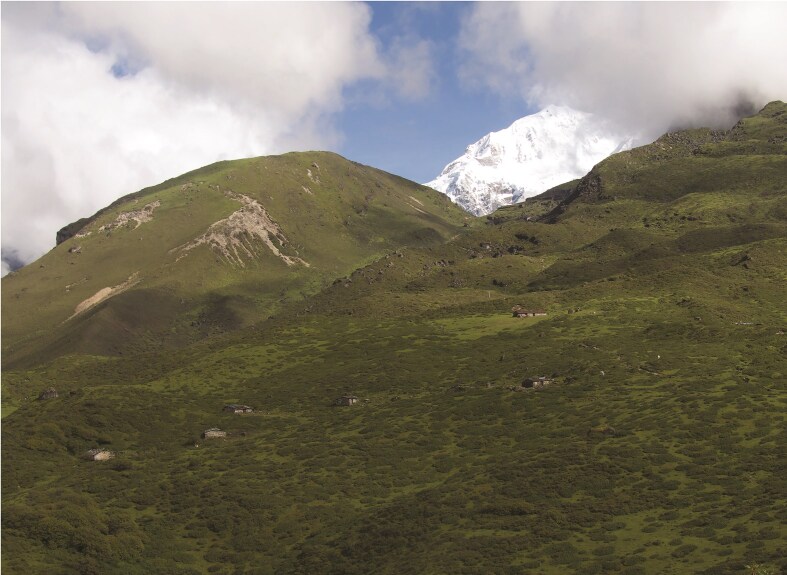
Temporary settlements, goaths, that pastoralists had used during summer grazing of sheep and yaks in Khangchendzonga National Park prior to the state-imposed grazing ban. Photograph: Rashmi Singh, Indian Institute of Technology, Hyderabad.

Governance systems and policies designed to address pastoral issues often impose adverse consequences on pastoralists’ livelihoods and rangeland sustainability. Policies prioritizing agricultural intensification, forced settlement of mobile pastoralists, pastoralist displacement from protected areas in the name of conservation, and land expropriation and privatization for development projects frequently undervalue the ecological and cultural importance of pastoral systems, leading to restrictions on mobility, loss of grazing lands, and fragmentation of critical resources (Reid et al. 2014, Nori and Scoones 2023). Conservation strategies that exclude pastoralists from protected areas often disrupt pastoral communities and erode their customary governance systems (box 4, figure 3; Goldman 2011). Moreover, the lack of recognition of customary institutions in formal governance frameworks has weakened community-led management of pastoral systems, further exacerbating fragmentation and degradation (Nori and Scoones 2023). It is critical that outdated misconceptions of pastoral impacts on biodiversity conservation be amended to create governance and policies that value and support pastoralism as a strategy for both biodiversity conservation and pastoral sustainability.

Box 4. **Pastoral displacement in Indian Himalaya increased social inequalities with questionable conservation benefits**

A state induced ban on grazing was implemented in Khangchendzonga National Park, which is a UNESCO World Heritage Site, in Sikkim, Indian Eastern Himalaya (Singh 2022). The ban emerged from the authoritarian “green governance” model of the leading political party in the Sikkim. Pastoralists, who had traditionally grazed yak and sheep across alpine and temperate pastures during the summer, were evicted between 2000–2002. The grazing ban was implemented on the basis of the assumption that grazing had caused ecological damage, but without systematic analysis or pastoral participation, and provisions were not made for pastoral compensation or alternative livelihoods. Post ban, the State encouraged opening the park to ecotourism, including the use of the pack animals to support excursions into the park. However, only a small number of elite pastoralists obtained exclusive grazing access to the park through powerful political connections, in spite of the grazing ban. This led to an increase in horses and *dzo* (yak-cattle crosses) to serve as pack animals and a doubling of the number of yaks owned by the elites. Consequently, 15 years following imposition of the grazing ban, vegetation composition and plant species diversity did not show substantial “improvement,” or “recovery” in either the alpine or temperate pastures (Singh 2022). Pastoral evictions resulted in the further impoverishment of weaker sections of the pastoral community while powerful pastoralists appropriated benefits from these conservation policies. Social conflicts also emerged within the local community and led to the emergence of new conservation challenges in the form of increased incidents of negative human–wildlife interactions. State engagement with regional pastoralists to leverage pastoralist knowledge and governance may have more effectively conserved rangeland biodiversity and maintained social well-being of regional communities than did displacement of pastoralists. See figure 3.

## Recommended action

Ambitious goals have been established by the Kunming–Montreal Global Biodiversity Framework, including a halt to extinctions of all threatened species and a tenfold reduction in the extinction rates of all species by 2050 (Pereira et al. 2024). We contend that the promotion of sustainable pastoralism on rangelands represents a critical mitigation strategy to support achievement of these goals. This strategy is focused on conservation of the vast reservoir of rangeland biodiversity, which is currently unrecognized and underprotected, as a form of working lands conservation to simultaneously mitigate biodiversity loss and enhance livelihoods of rural communities (Kremen and Merenlender 2018). Greater recognition and inclusion of pastoralism within global conservation frameworks, especially the Convention on Biodiversity, and multilateral organizations has great potential to increase achievement of global biodiversity targets (Lewin et al. 2024).

We recommend five broad strategies to increase recognition and to initiate action to leverage the potential benefits of rangeland pastoralism on biodiversity conservation. First, reshape the dominant conservation narrative from on eindicating that pastoralists are a threat to biodiversity and protected area management to one that envisions pastoralists as an asset for biodiversity conservation. Second, recognize that grazing and fire are natural ecological disturbances that maintain rangeland biodiversity and that humans have long used them to modify vegetation and improve forage quality. Third, develop policies and promote investments that incentivize and support pastoral practices synergistic with biodiversity conservation and minimize existing perverse policies and incentives, especially those that contribute to rangeland conversion and fragmentation. Fourth, redesign and implement regional infrastructure to support pastoral mobility and pastoralists’ development goals, including drove roads and other flexible movement corridors, market access, communication services, mobile education, and human and livestock health services, while increasing the sustainability and mobility of rangeland pastoralism. Fifth, support secure collective land and water tenure for pastoralists, adaptive, community-led rangeland governance, and effective and equitable institutions to provide pastoralists rights to self-determination, knowledge sovereignty, and conflict management to enhance security.

Transformation of societal perspectives and governance systems are required to envision and promote pastoralism as a vital conservation strategy (Briske and Coppock 2023, Kennedy et al. 2023). This will require a more expansive and inclusive view of area-based biodiversity conservation that recognizes key characteristics spanning area extent, ecological integrity, connectivity, and equitable governance (Robinson et al. 2024). Greater representation of local communities and local or Indigenous knowledge, particularly in the context of sustainable pastoralist practices, will also be essential (Kennedy et al. 2023). Synergies among pastoralism and biodiversity conservation provide critical opportunities to mitigate biodiversity loss through integration with existing protected areas to create a vast network for biodiversity conservation.

## Author Biography

David D. Briske (dbriske@tamu.edu); is affiliated with the Department of Ecology and Conservation Biology, Texas A&M University, in College Station, Texas, in the United States. Joris Cromsigt is affiliated with the Department of Wildlife, Fish, and Environmental Studies at the Swedish University of Agricultural Sciences, in Umeå, Sweden; with the Copernicus Institute of Sustainable Development, at Utrecht University, in Utrecht, in The Netherlands; and with the Centre for African Conservation Ecology, at Nelson Mandela University, in Gqeberha, South Africa. Jonathan Davies is an independent researcher in Hereford, Herefordshire, England, in the United Kingdom. María E. Fernández-Giménez is affiliated with the Department of Forest and Rangeland Stewardship at Colorado State University, in Fort Collins, Colorado, in the United States. Matthew W. Luizza is affiliated with the Natural Resource Ecology Laboratory at Colorado State University, in Fort Collins, Colorado, in the United States. Pablo Manzano is affiliated with the Basque Centre for Climate Change, in Leioa, Spain, and with Ikerbasque, the Basque Foundation for Science, in Bilbao, Spain. Rashmi Singh is affiliated with the Department of Liberal Arts at the Indian Institute of Technology, in Hyderabad, India
